# Expanding horizons: integrating multichannel deep learning predictors into patient-specific digital twins for precision lung cancer management

**DOI:** 10.1097/JS9.0000000000005064

**Published:** 2026-03-25

**Authors:** Ruixin Chu, Wenrui Wang

**Affiliations:** a Department of Pathogen Biology, Key Laboratory for Pathogen Infection and Control of Jiangsu Province, Nanjing Medical University, Nanjing, China; b Department of Thoracic Surgery, The First Affiliated Hospital of Bengbu Medical University, Bengbu, Anhui, China; c Anhui Provincial Key Laboratory of Tumor Evolution and Intelligent Diagnosis and Treatment, School of Life Sciences, Bengbu Medical University, Bengbu, China

*Dear Editor,*

We read with great interest the recent article by Geng *et al*, “Multichannel deep learning prediction of major pathological response after neoadjuvant immunochemotherapy in lung cancer: a multicenter diagnostic study”[1]. The authors present a compelling multichannel deep learning framework, Transformer_GoogLeNet, integrating preoperative CT imaging across multiple anatomical planes via Transformer-based fusion, achieving exceptional diagnostic accuracy (AUC = 0.924) for predicting major pathological response (MPR) in non-small cell lung cancer (NSCLC) patients undergoing neoadjuvant immunochemotherapy (NAICT). This represents a significant technical advancement in leveraging artificial intelligence (AI) for pretreatment prognostication. While the study thoroughly validates the predictor’s immediate diagnostic performance, we wish to expand the conversation toward its long-term clinical integration potential within evolving digital health ecosystems, specifically digital twins (DTs).

The Transformer_GoogLeNet model effectively translates static, pretreatment CT data into a predictive computational phenotype. Integrating such models within longitudinal digital twin frameworks could dramatically amplify their clinical utility beyond a single timepoint assessment. DTs are virtual, dynamic representations of individual patients, continuously updated with multi-modal data streams throughout their health journey[2]. Embedding the validated multichannel deep learning model as a core predictive component within a NSCLC patient’s DT would create a “living prognostic signature.” This digital twin could assimilate serial data, including subsequent imaging assessments, molecular biomarkers (e.g., circulating tumor DNA), treatment responses, and patient-reported outcomes, continuously refining the MPR probability and enabling adaptive surgical planning guidance as the treatment course unfolds[3]. This dynamic, closed-loop prognostication could offer a more nuanced strategy compared to solely relying on the pre-treatment snapshot.

Furthermore, the development pipeline established by Geng *et al*, particularly the multichannel Transformer-based fusion approach adept at integrating diverse, high-dimensional imaging datasets, provides a valuable template for building comprehensive DT simulators. The future extension of this methodology to incorporate serial radiomics trajectories, multi-omic features, and wearable sensor data could populate complex DT models. These models could serve as computational “sandboxes,” enabling clinicians to simulate individual patient responses to alternative NAICT regimens or surgical strategies prior to clinical decision-making[4]. Such predictive simulation aligns directly with the core tenets of precision oncology, aiming to forecast patient-specific outcomes and optimize therapeutic pathways.

In conclusion, we applaud Geng *et al* for their rigorous methodology and validation, establishing a robust foundation for pre-treatment MPR prediction[1]. We suggest that the natural progression of such sophisticated predictive biomarkers lies in their incorporation into broader digital health strategies like digital twins. This evolution demands focused research on secure, interoperable data integration platforms enabling continuous model updating within clinical workflows. Prospective validation of dynamic DT-guided decision-making against standard care will be essential to demonstrate tangible improvements in treatment efficacy, surgical outcomes, and quality of life. Collectively, this valuable work provides a critical component for realizing this exciting horizon in AI-enhanced, patient-centered oncology. We ensure this article is compliant with the TITAN Guidelines[5].

## Data Availability

Not applicable.
